# Supplementary material for: Soluble triggering receptor expressed on myeloid cells 1 is associated with hemoconcentration and endothelial activation in children and young adults with dengue virus infection in the Philippines
Source: PLoS Negl Trop Dis. 2025 Jun 2;19(6):e0013084. doi: 10.1371/journal.pntd.0013084 (PMC12180410; doi:10.1371/journal.pntd.0013084)
Supplement: Attachment — Submitted filename: Reviewer recommendations.pdf [file pntd.0013084.s002.pdf]

REVIEWER RECOMMENDATIONS

The study analises the relation between sTREM-1 and the progression of the dengue virus infection in 244 Philippines children and young patients, due to its role as a marker of neutrophils activation. It describes a significative association of sTREM-1 high levels with the hemoconcentration, and also with high levels of inflammation markers (WBC, neutrophils, TNF, Ang2, sFlt-1, sVCAM-1). The authors propose sTREM-1 as a marker of severe progression in dengue virus infection and that could facilitate the identification of those patients at risk.

Review Questions

Methods:

- The authors have written the *Study Procedures* at *Methods*, describing *Venipuncture blood was collected for complete blood count*, nevertheless, the chronological point during the infection course when the venipuncture was realised is not defined. Furthermore, at the last paragraph of the *Introduction*, the authors have written *Our primary objective was to investigate the association between sTREM-1 and subsequent hemoconcentration*, however, it is not explained when they have calculated the hemoconcentration. I think that including a further description of the chronology of the samples extraction should be essential for a better understanding of the whole manuscript.
- The authors have indicated at *Study Procedures* that *Participants were followed-up after resolution of the acute illness (day 14-21 after presentation) to review and document their clinical course, including hospitalization and intravenous fluid management*, in spite of that, they have not included in Table 1 or in another table which characteristics or symptons they collected at that point. Moreover, they have not included data about hospitalization at Table 1, unless that *Warning signs* and *Severe dengue* indicated hospitalization, even so, I think that could be appreciated to add or clarify all this information.
- *Minor point:* The authors should include the number of patients included at *Study Design and Participants*.

Results:

- I think that the figures designed for the Results are really accurate and show the data in an easy way to understand. However, I suggest to develop somewhat the results, showing the marker values, in order to help other researchers in their studies of endothelial activation.

Conclusions:

- The conclusions are supported by the fact that the sTREM-1 high levels are significantly associated with the hemoconcentration and the high levels of inflammation markers (WBC, neutrophils, TNF, Ang2, sFlt-1, sVCAM-1). It has allowed the authors to propose sTREM-1 as a marker of severe progression in dengue virus infection and that could facilitate the identification of those patients at risk. However, the title of the manuscript may not seem the most suitable with the conclusions presented at the end of the Discussion ("sTREM-1 was predictive of hemoconcentration"). Taking this sentece as the main conclusion, I consider that it could be interesting to draw the title up again from another point of view.
- The limitations of analysis are clearly described at the Discussion.

- Furthermore, these findings could have clinical implications as they mentioned in the Discussion: "Integrating sTREM-1 into rapid diagnostic tests could facilitate the identification of at-risk patients and those who can be safely managed as outpatients".

Editorial and Data Presentation Modifications:

The authors have analysed the values from children and young patients, but it seems that they have not analysed those data separately. From my point of view, I think that it could be useful to do that analysis in order to observe any differences in the correlation of the markers between children and young patients, or even between children and teenagers with young patients, due to the fact that the immune system is developing until the adolescence.
In addition:
- The authors do not mention where they have realised the Hematologic parameters included in Table, I may recommend them to do it.
- The authors have described the association between sTREM-1 and TNF but they have not included a figure with that information, I think that it would be appealing to add it.

Summary and General Comments:

One of the strengths of this study is the large cohort which includes molecular and clinical collected data. On the other hand, the authors have made an introduction of dengue fever and its incidence, but the context explanation of the disease around the world looks a bit short. It could be convenient to extend partially the introduction in order to justify the study and to highlight its originality, maybe it could be useful to add some references in that part. I may recommend to add as a reference the following review:

Clinical predictors of severe dengue: a systematic review and meta-analysis – Tsheten Tsheten, Archie C.A. Clements, Darren J. Gray, Ripon K. Adhikary, Luis Furuya-Kanamori and Kinley Wangdi. Infect Dis Poverty (2021).
