# Supplementary material for: Soluble triggering receptor expressed on myeloid cells 1 is associated with hemoconcentration and endothelial activation in children and young adults with dengue virus infection in the Philippines
Source: PLoS Negl Trop Dis. 2025 Jun 2;19(6):e0013084. doi: 10.1371/journal.pntd.0013084 (PMC12180410; doi:10.1371/journal.pntd.0013084)
Supplement: Attachment — Submitted filename: sTREM-1 PNTD review final.docx [file pntd.0013084.s003.docx]

**Soluble triggering receptor expressed on myeloid cells 1 predicts vascular leak in children and young adults with dengue virus infection in the Philippines**

This manuscript by Yang et al. explores the relationship between the soluble form of Triggering Receptor Expressed on Myeloid Cells 1 (sTREM-1) and hemoconcentration, a hallmark of vascular leakage in dengue. Previous studies have investigated sTREM-1 as a potential predictor of severe dengue, particularly when combined with other inflammatory markers.

In their study, Yang et al. analyzed 244 children and young adults (aged 1–26 years), measuring sTREM-1 levels using a multiplex Luminex® assay. They assessed the correlation between sTREM-1 levels and the development of hemoconcentration, defined as an increase of >20% from baseline hematocrit. Additionally, the authors examined the association between hemoconcentration and the risk of hospitalization, as well as the need for fluid resuscitation. Their findings indicate a significant association between elevated sTREM-1 levels and hemoconcentration, and between hemoconcentration and increased hospitalization risk and higher fluid management requirements.

However, the study's main limitation lies in the lack of evidence demonstrating that elevated sTREM-1 levels precede the onset of hemoconcentration. This omission undermines the primary claim in the title, “Soluble Triggering Receptor Expressed on Myeloid Cells 1 Predicts Vascular Leak in Children and Young Adults with Dengue Virus Infection,” as the predictive role of sTREM-1 cannot be conclusively established without temporal data showing that the exposure (sTREM-1 levels) was present before the outcome (hemoconcentration). Moreover, the authors show that sTREM-1 levels were also associated with sex (Table 1), indicating the need to address potential confounders in a multivariate analysis.

**Major comments:**

1. **Predictive value of sTREM-1:**
   - The manuscript lacks clarity on the time points for sample collection and outcome development. These details are critical for establishing the predictive value of sTREM-1 levels, as claimed by the authors in the title. To claim prediction, it is imperative to provide evidence that the exposure (sTREM-1 levels) was present at a date before the outcome (hemoconcentration) was measured. Otherwise, the study shows association not prediction.
2. **Data analysis:**
   - The authors should conduct a logistic regression analysis to assess the relationship between sTREM-1 levels (quantitative) and the odds of developing hemoconcentration. This would strengthen the evidence for the predictive role of sTREM-1. Based on the data on Table 1, it appears that sex is a potential confounder in the analysis. The authors should adjust by this variable in a multivariate analysis.
   - Given that based on what is currently reported in the study, it is difficult to establish if the exposure was present before the development of the outcome, a more appropriate measurement of the strength of association would be the Odds Ratio, instead of the Relative Risk.
3. **Variable correlations:**
   - The authors should show the correlations between white blood cell count, neutrophils, platelet, lymphocytes, Ang-2, sVCAM-1, and sFlt-1 with the quantitative values of sTREM-1 (instead of its categorial values <130pg/mL, ≥130pg/mL). These analyses would provide a more comprehensive understanding of the data.

**Minor comments:**

1. Ensure compliance with the STROBE (Strengthening the Reporting of Observational Studies in Epidemiology) guidelines.
2. The subheading: “Elevated sTREM-1 is associated with endothelial activation” should be to a more precise statement “Elevated sTREM-1 is associated with markers of endothelial activation”
