# Supplementary material for: Soluble triggering receptor expressed on myeloid cells 1 is associated with hemoconcentration and endothelial activation in children and young adults with dengue virus infection in the Philippines
Source: PLoS Negl Trop Dis. 2025 Jun 2;19(6):e0013084. doi: 10.1371/journal.pntd.0013084 (PMC12180410; doi:10.1371/journal.pntd.0013084)
Supplement: Attachment — Submitted filename: Response to editorial changes Yang sTREM-1.docx [file pntd.0013084.s005.docx]

28 March 2025

Dear Nithya Venkatesan and Editorial Team,

Thank you for the Technical Check of our revised manuscript entitled “*Soluble triggering receptor expressed on myeloid cells 1 is associated with hemoconcentration and endothelial activation in children and young adults with dengue virus infection in the Philippines.*” The following changes have been implemented in a revised manuscript. We have uploaded the revised manuscript using the online portal.

**1) The following file is currently uploaded as file type 'Other', which is not viewable by the reviewers: sTREM1 Data.xlsx. Please change the file type to 'Supporting Information' and include a legend in the manuscript if you wish it to be included in review.**

We have uploaded the excel file as Supporting Information and included a legend in the manuscript. We have uploaded this as a .zip file.

**2) Thank you for stating "The authors declare that they have no known competing financial interests or personal relationships that could have appeared to influence the work reported in this paper."**

**Please include a completed 'Competing Interests' statement in the text box when submitting your production task, including any COIs declared by your co-authors, written in full sentences.**

**If you have no competing interests to declare, please state "The authors have declared that no competing interests exist". You may also provide an updated statement via email.**

Thank you. We have modified the sentence in the Competing Interests text box as follows:

“The authors have declared that no competing interests exist.”

**3) Please ensure that the affiliations of the authors (Jiayi Yang, Maria Salome Siose Painaga, James Yared Gaite, Ashley Roberts, Kevin C. Kain, Michael T. Hawkes] listed on the manuscript title page do exactly match with the affiliations provided in the online submission form**

**NOTE: Affiliations should include a department (if applicable), an institution, a CITY, and a country**

Thank you. We have added a city for authors on the online submission form and a Department where this information was missing.

**4) We do not publish any copyright or trademark symbols that usually accompany proprietary names, eg ©, ®, or TM (e.g. next to drug or reagent names). Therefore please remove all instances of trademark/copyright symbols throughout the text, including: Bioline™ on page no 6.**

Thank you. We have removed the trademark symbol on page 6. We searched the article and we did not find any other instances of copyright or trademark symbols.

**5) Thank you for stating "Written informed consent was obtained from all participants or caregivers."**

**If child participants, the statement must declare that formal consent ( must state whether verbal/written)was obtained from the parent/guardian.**

Thank you. We have modified the statement as follows: “Written informed consent was obtained from participants or, in the case of children, from the parent or guardian.”

**6) Please upload all main figures as separate Figure files in .tif or .eps format. For more information about how to convert and format your figure files please see our guidelines:**

**https://journals.plos.org/plosntds/s/figures**

Thank you. We have uploaded the figures as .eps files.

**7) In the online submission form you indicate that your data is not available for proprietary reasons and have provided a contact point for accessing this data. Please note that your current contact point is a co-author on this manuscript. According to our Data Policy, the contact point must not be an author on the manuscript and must be an institutional contact, ideally not an individual. Please revise your data statement to a non-author institutional point of contact, such as a data access or ethics committee, and send this to us via return email. Please also include contact information for the third party organization, and please include the full citation of where the data can be found.**

Thank you. We have modified the Data Availability statement as follows: “Raw data used for the analysis has been included as Supporting Information (excel file). Additional information can be obtained from the corresponding author, upon reasonable request.”

**8) Please ensure that the funders and grant numbers match between the Financial Disclosure field and the Funding Information tab in your submission form. Note that the funders must be provided in the same order in both places as well.**

**Currently, " Canada Research Chair (KCK), and kind donations from Kim Kertland, and Rotary International (K-W group)" are missing from the Funding Information**

Thank you we have added CIHR and Rotary Foundation to the list of funders on the online submission form.

We thank the editorial team for the initial technical check of our manuscript. We look forward to your assessment of the revised manuscript.

Yours sincerely,

Dr. Michael Hawkes, MD, PhD

Associate Professor

Department of Pediatrics

Faculty of Medicine

University of British Columbia, Canada
